# Practical Outcomes From CASP16 for Users in Need of Biomolecular Structure Prediction

**DOI:** 10.1002/prot.70078

**Published:** 2025-10-15

**Authors:** Luciano A. Abriata, Matteo Dal Peraro

**Affiliations:** ^1^ Laboratory for Biomolecular Modeling and Protein Structure Core Facility, School of Life Sciences École Polytechnique Fédérale de Lausanne (EPFL) and Swiss Institute of Bioinformatics Lausanne Switzerland

**Keywords:** alphafold, molecular modeling, structure prediction

## Abstract

The 16th Critical Assessment of Structure Prediction benchmarked advancements in biomolecular modeling, particularly in the context of AlphaFold 2 and 3 systems. Protein monomer and domain prediction is largely solved, with barely any space for further improvements at the backbone level except for very specific details, irregular secondary structures, and mutational effects that remain challenging to predict. For protein assemblies, AF‐based methods, especially when expertly guided or enhanced by servers like those from the Yang, Zheng/Zhang, and Cheng lab, show progress, though complex topologies and in particular antibody–antigen interactions are still difficult. Notably, a priori knowledge of stoichiometry significantly aids assembly prediction. Protein‐ligand co‐folding with AF3 demonstrated strong potential for pose prediction, outperforming many participants and some dedicated docking tools in baseline tests, but several caveats hold as discussed. Ligand affinity prediction is totally unreliable. Nucleic acid structure prediction lags considerably, heavily relying on 3D templates and expert human intervention, even AF3 showing substantial limitations. Overall, on all fronts, AF3's modeling capabilities are at or close to the state of the art; additionally, it shows slight improvements over AF2 and more detailed confidence metrics than it. We guide users on tool selection, realistic accuracy expectations, and persistent challenges, emphasizing the critical role of confidence metrics in interpreting AI‐generated models.

## 1 Introduction: Context and Some Important Definitions

For over 30 years, the Critical Assessment of protein Structure Prediction (CASP) experiment has served as a crucial community‐wide benchmark for biomolecular structure prediction [1], with major recent breakthroughs that quickly unlocked progress in other fields and eventually impacted all biology [2, 3]. CASP challenges predictors to model the 3D structures of biomolecules and their complexes before their experimental structures are publicly released, providing an objective assessment of the state of the art. Each edition has tracked the evolution of prediction methodologies, from early homology modeling to the deep learning‐driven breakthroughs [4, 5, 6] materialized in AlphaFold 2 (AF2) [7, 8]. CASP15 witnessed the widespread adoption of AF2‐like systems and started to stress focus on more complex targets, including multimeric assemblies and non‐protein components [9, 10, 11].

During CASP16 (2024), AlphaFold 3 (AF3) [11] became available with its capability to model a wide range of biomolecules to include not only proteins but also nucleic acids, ions, and small molecules, just like RoseTTAFold‐AllAtoms [10] released a few months before it and models from the Boltz [9, 12] and Chai [13] families that followed soon after, among others. This was perfect timing for CASP16, which in its nine broad modeling categories ranging from protein monomers and multimers to complexes included the largest‐ever benchmarks on nucleic acids and on small molecule ligands, the latter especially enticing as it dealt with curated cases of pharmacological relevance.

**FIGURE 1:**
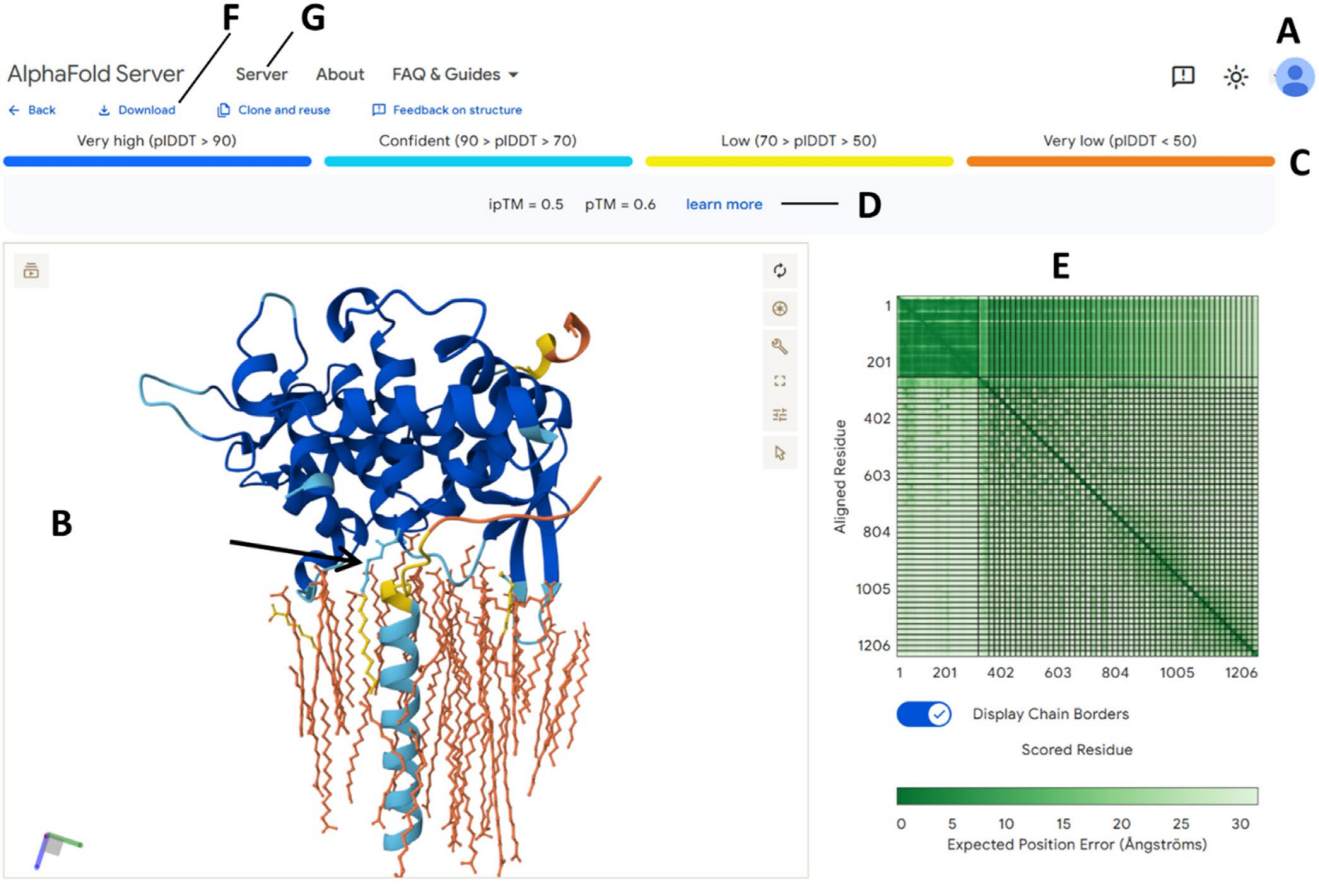
AlphaFold 3 server workflow for modeling an assembly and details on its confidence metrics. (A) A valid Google account is needed to use the AF3 server at https://alphafoldserver.com/. Make sure you get familiar with its license, which does not allow commercial use (among other limitations; see text and Table 1) as of the date of acceptance of this paper. (B) Example 3D model for a complex between a peripheral membrane protein with a palmitoylated cysteine (arrow), a short integral membrane helical protein, and 50 lipid molecules that AF3 assembled into a bilayer‐like structure that reflects the true nature of this complex. More specifically, this is Cys84‐palmitoylated GOLPH3 bound to the LCS peptide, a system explored through integrative modeling by Theodoropoulou et al. [19] (C) Color scale for pLDDT, a per‐atom confidence estimate on a 0–100 scale where higher values indicate higher confidence. The pLDDT is color‐coded onto the structure outputs (B) using the same mapping used in the AFDB [20]; proteins are colored by residue‐wise pLDDT while non‐standard moieties like the cysteine's palmitate group are colored by atom‐wise pLDDT, as the atoms of small molecule ligands would be colored by default as well. Note that in the CIF output, all atoms are flagged with atom‐wise pLDDT on the B‐factor fields, allowing easy visualization in programs like PyMOL (Figure S1). (D) The global scores pTM and ipTM act as single‐number metrics for overall model confidence (pTM) and for confidence of all interfaces (ipTM). The predicted template modeling (pTM) score and the interface predicted template modeling (ipTM) are derived from the template modeling (TM) score long‐utilized in CASP [21]. A pTM score above 0.5 means the overall predicted fold for the complex might be similar to the true structure. ipTM measures the accuracy of the predicted relative positions of the subunits within the complex. As explained in the official AF3 server, pTM and ipTM values higher than 0.8 represent confident high‐quality predictions, while values below 0.6 denote poor predictions—but see Figure 2 for a deeper example. Note that while ipTM attempts to quantify the quality of all interfaces in a single number, the full AF3 outputs also include ipTM values for all possible pairs of chains, which allows one to more narrowly tell which interfaces might be well predicted and which ones probably are not. For details and a discussion on some potential problems with ipTM scores, the reader is referred to a dedicated study by Dunbrack [22]. (E) The pAE plot quantifies how reliably each residue was positioned relative to all others in the model. Here, lower (greener) is better. The pAE plot is useful to gauge how well domains are placed within proteins, and how well pairs of molecules are placed within complexes. (F) On download, the user obtains the full data required to reproduce these and other graphics (see Figure S1 and https://go.epfl.ch/af3scores) as well as 5 models (the one shown on the webpage is the one that AF3 ranked as best). (G) This whole figure refers to the server version of AF3, but for full capabilities as discussed in the text (custom templates and MSAs, access to all ligands, etc.) the user should get a local installation following https://github.com/google‐deepmind/alphafold3. (H) For more details about the AF3 server, including more information about the confidence scores, the reader is referred to https://alphafoldserver.com/faq.

In this article, we distill the practical outcomes from CASP16, aiming at end‐users seeking to apply biomolecular structure prediction software to their research. A dominant theme in CASP16 was the central role of AF‐based approaches, either used directly or as part of other pipelines, with CASP adopting regular AF2 via ColabFold and AF3 from the official server implementation as baselines, the latter dominant and emerging as the main recommendation (Figure 1). Here we focus on identifying which methods and approaches performed best across different prediction categories, if and how they improve over AF2 and AF3, their general availability, realistic expectations of accuracy, and current limitations, summarizing the comprehensive observations reported by the CASP16 assessors in a practical format with simplified jargon [14, 15, 16, 17, 18]. Sections 2, 5 cover recommendations for modeling different kinds of biomolecules as separately assessed in different CASP16 tracks, while Section 6 provides a single‐shot summary of CASP16's recommendations for biomolecular structure prediction in general. Throughout the whole review, we stress the role of various confidence scores.

Importantly, CASP involves both “human groups,” who apply manual intervention and expert knowledge, and “server groups,” which act as fully automated methods, some of which become accessible online after CASP. Typically, human groups perform as well as or better than servers, and all CASP editions have consistently found that predictors are not very good at scoring their or other predictors' models, even when among the models there are some that match the true structure perfectly. The assessors' papers deal with both kinds of predictor groups, drawing only a partial distinction between those that stand as resources that end‐users can actually use, those that involve closed pipelines that are in development, and those that participated as human experts. Here, we will focus largely on those that ranked near the top and exist as actual tools for users to easily utilize (links in Table 1), barely mentioning some very salient findings on human predictors.

**TABLE 1:** URLs for CASP16 baseline methods and top servers.

Server/Software (References)	URL	Advantages over AF3 server	Notes
*Baselines*			
AlphaFold 3 website [11]	https://alphafoldserver.com	NA (baseline)	Limited number of submissions per day; no custom template or MSA; limited set of ligands.
AlphaFold 3 code [11]	https://github.com/google‐DeepMind/alphafold3 https://github.com/google‐DeepMind/alphafold3/blob/main/docs/input.md	Full control on templates and MSAs; any small molecule can be inputted. Easier to sample more by repeating runs with different seeds. Larger systems might be fit than in the server, if modern GPUs are used. Number of runs only limited by user's access to hardware resources.	The second link is the official description of all inputs. For online inspection of quality scores, check https://go.epfl.ch/af3scores.
AlphaFold 2 via ColabFold [7, 8, 23, 24]	https://github.com/sokrypton/ColabFold	Full control on templates and MSAs.	“AlphaFold2_mmseqs2” notebook recommended, which automatically uses AF2‐multimer if needed. Google Colab Notebook needs some effort to use and depends on cores being available. Limited number of runs per day on free Colab plan.
*Methods that ran in automated fashion for CASP16 (“servers” with their official CASP16 names)*			
MIEnsembles‐Server [25]	https://seq2fun.dcmb.med.umich.edu/MIEnsembles‐Server/	Automatic stoichiometry prediction. Potentially slightly better results than AF3 for complexes.	Providing models runs scoring and ranking.
Yang‐Server and Yang‐Multimer https://onlinelibrary.wiley.com/doi/10.1002/prot.70030	https://yanglab.qd.sdu.edu.cn/trRosetta/	Potentially slightly better results than AF3 for complexes.	CASP16 version will be put up late 2025.
MULTICOM_X [26]	https://github.com/BioinfoMachineLearning/MULTICOM4	Potentially slightly better results than AF3 for complexes.	MULTICOM4 is the structure prediction engine for monomers and multimers, while the various MULTICOM_X programs rank and select models in different ways.

## 2 Modeling of Protein Domains and Whole Monomeric Proteins

### 2.1 What's Currently Possible and What Isn't?

CASP16 results indicate that single‐domain protein fold prediction is nearly solved, with successful modeling across all defined evaluation units [17]. This success is largely due to the continued refinement of AlphaFold‐based pipelines. However, it is important to note that this achievement is primarily for targets with at least distant homology to known structures. Scientists working on truly novel proteins with no detectable homology should proceed with caution. For proteins with very shallow MSAs, such as viral and some eukaryotic proteins, modeling might be more challenging.

While the overall fold may be correct, high local accuracy (e.g., side‐chain conformations, loop regions) is not guaranteed. Improvement in these aspects from CASP15 to CASP16 was subtle. Challenges remain especially in the modeling of truncated sequences, irregular secondary structures such as bent helices, conformations induced by interchain interactions (for monomers extracted from complexes), uncommon isomers, effects of single or few mutations, or large regions with irregular structures. The paper assessing monomer modeling shows examples of these kinds of failures in its figure 3 [17].

### 2.2 Recommended Servers and Their Advantages Over AF3

Servers from the Yang lab, the Zheng/Zhang lab, and the Cheng lab achieved top performance across monomer targets, slightly above AF3. AF3, as a web server, demonstrated a small but noticeable advantage over AF2, particularly in its confidence estimation and model selection capabilities. However, and very notably, when ranked by the quality of “model 1” submissions at the domain level (in CASP, each predictor typically submits 5 models), the AF3 server itself rose to second place, being essentially indistinguishable from other top groups, even human experts. Overall, then, our recommendation is here on AF3. We further note that the ColabFold implementation of AF2 ranked well behind (see figure 6A of the monomer assessment paper [17], where the ColabFold baseline is not ranked).

It is important at this point to note that CASP rankings based on Z‐scores amplify small differences, and the absolute quality differences between top methods and baseline ColabFold or AF3 when compared by TM or GDTTS scores are very small, just marginal for well‐behaved domains.

### 2.3 Tips and Tricks From Top‐Performing Groups

Top‐performing groups capitalized on AF2 and AF3 by employing strategies such as meticulous optimization of MSAs by increasing depth (number of sequences) and/or semi‐manually curating them, careful selection of “constructs” (the specific protein sequence fragment used for modeling), and enhanced conformational sampling with AF2 and AF3 [26, 27] (an approach tested thoroughly in a dedicated part of CASP16 using MassiveFold [28, 29]). Unfortunately, strategies involving manual optimization of MSAs, templates, and constructs entail extensive manual intervention guided by human expertise and experience in modeling that naturally cannot be embodied in the software.

Of note and relevant to those running the different programs listed in Table 1, while deeper MSAs generally correlate with better accuracy, AF2 and AF3 can produce good models even with shallower or no MSAs for some targets. However, users should be aware that this isn't guaranteed for all proteins with very shallow MSAs; for example, many viral and some eukaryotic proteins performed rather poorly compared to the majority of targets which were very well modeled [17]. In our view, there is no sufficiently deep information about this in the CASP16 assessments, so we limit ourselves to make the reader aware of the potential relevance of MSAs, particularly when a prediction comes out with poor quality estimate scores.

Another conclusion from the monomer assessment paper was that for modeling portions of multi‐domain proteins, or proteins that are part of larger complexes, careful definition of the domain boundaries or the segment to be modeled is crucial. This involves “chopping” the target sequences in different places, checking by trial and error how to optimize the software‐provided confidence metrics, especially pLDDT and intrachain pAE when using AF2 or AF3.

Finally, it is important to keep in mind that model ranking (i.e., selecting the single best model from among multiple predictions) by all these programs remains a general weakness, and while the confidence metrics provide a good guide, they aren't infallible. Yet, confidence metrics offer valuable guidance, and when considering multiple models, especially if extensive sampling is used, they can help to narrow down the number of models that will be subject to deep expert inspection. Ultimately, users must leverage any experimental data and biological sense to guide modeling whenever possible, as well as to critically analyze the results in light of the confidence scores. For modeling domains and isolated proteins, the most important metrics to look at are pTM (or simply TM) as a global indicator, pLDDT for a per‐residue score, and pAE to assess the relative quality for each residue relative to all others (Figure 1).

## 3 Modeling of Protein Assemblies and Complexes

Predicting the structure of protein complexes (oligomers) is a frontier very important in fundamental and applied biology, on which CASP has been pushing for especially in the last three editions. CASP16 featured several protein‐only complexes and complexes of proteins with nucleic acids; some ligands were present in these complexes too, but separate assessments were carried out for these special kinds of complexes in a dedicated track as explained in the introduction and as detailed in a dedicated paper of this issue [30].

### 3.1 What's Currently Possible and What Isn't?

CASP16 results indicate that complex structure prediction remains an unsolved challenge, with 50% of the targets getting only partially good predictions and over 30% of the total targets proving highly difficult [15]. This difficulty is particularly evident for assemblies with novel interfaces lacking co‐evolutionary signals or template information, and for special cases such as antibody–antigen complexes, all of which occurred often in the CASP16 dataset making this track really difficult—and leaving hope for the simpler cases one may actually encounter. Nevertheless, moderate overall improvement was seen compared to CASP15. Very large or topologically intricate complexes, especially those with novel interfaces or lacking good templates, are still poorly predicted. Very large targets were also problematic.

Achieving high atomic accuracy at protein–protein interfaces (crucial for applications like interface‐targeted drug design or to design mutations that will disrupt a signal, etc.) remains a significant hurdle. Moreover, sometimes even when the models have a rather good overall score, the interfaces might be somewhat far from perfect, and vice versa. Part of this is due to far‐from‐perfect model quality estimation and model selection.

Notably, some kinds of complexes remain particularly difficult to model, such as Antibody–Antigen (AA) complexes and others with intricate topologies, even if not too large. Modeling protein‐nucleic acid (NA) complexes and interfaces was generally even more challenging than for the protein–protein case (detailed below in Section 3.4), partly due to the inherent difficulties in modeling nucleic acids (covered in Section 5).

### 3.2 Recommended Servers and Their Advantages Over AF3

Although most participating groups used AlphaFold2‐Multimer (AFM) or the AF3 server (the local version was not available while CASP16 rolled out) at the core of their modeling engines, the top‐performing groups of this track could significantly outperform default AFM/AF3 predictions by using optimized MSAs, refined constructs that allowed modeling large complexes in pieces, employing extensive model sampling, and applying specialized model selection techniques.

Among servers or downloadable software that participated as automated methods in CASP16, those ranked above AF3 include some of the MULTICOM servers, Yang‐Multimer and Yang‐Server, and the MIEnsembles‐Server, all linked in Table 1. These servers showed a small but sizable improvement over AF3, as shown in figure 3 of the paper assessing protein multimer modeling [15]. Meanwhile, servers and software like RoseTTAFold‐AllAtoms, Chai and Boltz did not participate and were not tested as baselines in CASP16, so any performance claims lack the rigor of a CASP‐level blind study.

### 3.3 Tips and Tricks From Top‐Performing Groups

A first important point to stress is that knowing the true stoichiometry beforehand when predicting the structure of a complex virtually always improves the accuracy of predicted assemblies quite significantly. It turns out that CASP16 began with what was called “Phase 0” in which the sequences of the molecules involved in the complex were provided but not the stoichiometries, and was followed by “Phase 1” where predictors were also given the stoichiometries. This experiment clearly demonstrated the importance of encoding the correct stoichiometric information (detailed by the assessors in figure 5 of their paper [15]) and implies that users should try to leverage experimental data (e.g., SEC‐MALS, native mass spectrometry) to guide modeling whenever possible.

Next, as discussed above, deeper, expert‐curated MSAs can help to boost predictions, meaning that it is worth spending time on this specific task. Unfortunately, though this is not applicable in any of the servers given in Table 1, as they do not allow users to upload custom MSAs. This is, however, possible on AF2 run via ColabFold and in locally installed versions of AF3.

Among specific challenges, it was clear in CASP16 that very large or topologically intricate complexes, especially those with novel interfaces or lacking good templates, are still poorly predicted. In such cases, strategies like “divide and conquer” modeling subcomplexes or domains separately and then assembling them were common and worked in some but not all cases. Unfortunately, though there are no clear guidelines on how to best split targets, CASP has not investigated this in detail. Notably, some kinds of complexes remain particularly difficult to model, such as Antibody–Antigen complexes and even if not too large. The kozakovvajda group, using a pipeline based on their ClusPro docking server [31] augmented with other techniques rather than relying mainly on AFM/AF3 predictions, significantly outperformed other groups, including extensively sampled AF3, on Antibody–Antigen targets (see figure 4 of the paper assessing protein multimer modeling [15]). Unfortunately, these participants were human only; hence, these methods are not available off‐the‐shelf online as of mid 2025.

As an additional recommendation, a recent study found that when modeling protein complexes with AF3 it is crucial to consider contextual elements (e.g., correct inclusion of ions, ligands, cofactors) in order to achieve the most accurate modeling possible for protein complexes [32].

To conclude this section, CASP16 made it clear that achieving high atomic accuracy at protein–protein interfaces (crucial for applications like interface‐targeted drug design or to design mutations that will disrupt a signal, etc.) remains a significant hurdle. Moreover, sometimes even when the models have a rather good overall score, the interfaces might be somewhat far from perfect, and *vice versa* [15]. Unfortunately, part of this is due to far‐from‐perfect model quality estimation and model selection. To assist these modeling tasks, interface confidence scores such as AF3's pTM and ipTM (interface‐specific pTM) are very useful together with the pAE plots (useful to assess relative domain and subunit placement), yet not infallible. A strategy that might work involves running the prediction protocols with multiple seeds to generate structural variability to be considered downstream in the framework of what is already known, expected, or more sensible for the system. Yet, as mentioned earlier, a meaningful biological interpretation is critical, as well as the use of any external data that can help decide among multiple models.

### 3.4 Modeling Hybrid, Protein‐Nucleic Acid Complexes

CASP16 featured an increased number of “hybrid” targets containing both proteins and nucleic acids (DNA/RNA). These were assessed by both multimer and nucleic acid assessors.

From the perspective of protein assembly assessment [15], the top‐performing groups for the protein–NA interfaces included mainly human predictors, plus some of the automated systems listed in Table 1. From a very practical point of view, among the servers and software evaluated in CASP16, only AF3 can natively model proteins and nucleic acids together in a single shot. Ideally, alternatives like RoseTTAFold‐AllAtoms, Chai, and Boltz models should be evaluated in the next round, either with their developers participating or as automated baselines just like CASP did for AF3 in CASP16.

The baseline AF3 server ranked further behind the specialized human groups for these hybrid protein‐nucleic acid targets. Overall, modeling protein‐nucleic acid complexes and interfaces was generally even more challenging than for the protein–protein case, including for the AF3 server. Part of this is naturally due to the inherently difficult nature of nucleic acid predictions, which, as detailed in the dedicated Section 5, lags much behind that of protein structure prediction. Moreover, tools like AF3 accept protein structures as templates but not nucleic acid structures, complicating even predictions that should presumably be on the easier side.

## 4 Modeling of Protein‐Ligand Complexes and Their Affinities

A very interesting highlight of CASP16 was a dedicated track for predicting protein‐small molecule (ligand) interactions, focusing on pharmaceutically relevant drug‐like compounds and involving both pose prediction (3D structure of the complex) and binding affinity prediction [16]. This track, made possible by collaborations with pharmaceutical companies (Hoffmann‐La Roche, Idorsia Pharmaceuticals) and the Structural Genomics Consortium, represented the most pharma‐relevant ligand prediction challenge in CASP history. Despite being the most serious test along these lines even in CASP, it must be noted that the dataset included only a handful of proteins in 229 protein‐ligand target structures (details on the dataset are provided by Tosstorff et al. [33]).

The main part of the assessment, briefly summarized here, focused on the drug‐like ligands present in the dataset, all with binding sites and poses well defined in the 3D structures, and in many cases also counting with affinities determined as part of industrial drug discovery projects. We touch here on the 3D modeling and affinity predictions for these ligands. Separately, the assessment paper [16] analyzes modeling of “incidental” ligands coming from cofactors, crystallization agents, etc., but we don't comment on them here. Besides, we touch on two important post‐CASP16 evaluations of ligand binding pose prediction with modern AI systems, mainly AF3 [34, 35].

### 4.1 Protein‐Ligand Pose Prediction

#### 4.1.1 What's Currently Possible and What Isn't?

For predicting the binding pose of drug‐like ligands, template‐based methods performed well, but only when templates were available. However, larger ligands (i.e., containing more atoms), more flexible ligands (containing more rotatable bonds), and ligands less similar to small molecules present in the PBD all show negative correlation with prediction accuracy in the CASP16 pharma dataset.

Despite AF3's apparently good performance in this area as evaluated in CASP16, two independent broader studies showed that the program relies largely on memorization, without much capability to understand the actual protein‐ligand interactions or doing good predictions for systems that are too far from those used in training [34, 35]. Notably, the limitations are stronger for ligands that have only been seen binding in one pocket, whereas more promiscuous ligands such as cofactors show moderately improved performance. This would mean that AF3 is much safer as a tool to model protein‐ligand complexes when reasonable templates exist in the PDB; and together with the various other caveats it is clear that co‐folding must for the moment be exercised with caution.

#### 4.1.2 Recommended Servers and Their Advantages Over AF3

Post‐CASP16, the assessors ran a set of automated baseline methods that were not available to participants during the challenge [16]. Strikingly, AF3 run locally as a co‐folding method (i.e., in which protein sequence and ligand SMILES are input together for concurrent modeling) achieved a mean LDDT‐PLI of 0.80, outperforming all CASP16 participating groups. Boltz‐1 and RoseTTAFold‐AllAtoms performed well behind AF3, with mean LDDT‐PLI values of 0.52 and 0.37, respectively. In one particularly remarkable case, AF3 predicted very accurately the pose of a ligand bound to a protein even though there's no similar binding site or close structural homologues in the PDB.

Note however that many leading developers of widely used academic and commercial docking suites (AutoDock Vina tested by CASP plus regular AutoDock, Glide, etc.) did not participate in this CASP16 challenge, making direct comparisons to the full spectrum of established methods difficult, and certainly leaving place for experts in these docking‐specific tools to perform much better than CASP's naïve runs.

#### 4.1.3 Tips and Tricks From Top‐Performing Groups

The “co‐folding” approach possible with structure prediction systems like AF3, where the protein and ligand are modeled simultaneously allowing for mutual conformational adaptation, could provide a key advantage over traditional docking software where the protein's structure must be known (or modeled) beforehand and is often treated as rigid or, in the best case, semi‐flexible. Note that a local AF3 installation is required in order to really access modeling of any ligand.

However, as discussed, these methods still need more benchmarking. Therefore, AF3 is much safer as a tool to model protein‐ligand complexes when reasonable templates exist in the PDB. Co‐folding in the absence of templates or for cases too far from those available in the PDB by the time of AF3's training must be exercised with caution. In any case, ipTM and pAE metrics can help gauge the accuracy of a ligand bound inside a pocket, together with the atom‐specific pLDDT introduced in AF3 that would report on the self‐estimated accuracy of the placement of each atom in the small molecule and also of the sidechains stabilizing it in the pocket.

Finally, given how the baseline methods (Autodock Vina, Glide, etc.) were used in CASP16, it cannot be ruled out that they actually could perform much better than presented, possibly even better than AF3 had they been executed by experts who master all their options and tricks.

### 4.2 Protein‐Ligand Affinity Prediction

Predicting binding affinities proved extremely challenging, with very poor correlation with the experimentally measured values [16]. Moreover, providing the actual experimental protein‐ligand complex structures to predictors in a second stage did not improve affinity prediction accuracy. This strongly suggests that the primary limitation lies in the scoring functions used by current methods, rather than in inaccuracies in the predicted poses themselves.

Simple ligand descriptors like molecular weight correlated with experimental affinities as well as, or sometimes better than, sophisticated computational methods. Docking scores from programs like AutoDock Vina or GNINA were not good predictors of affinity. It should be acknowledged, however, that standard docking scores are generally not optimized for, nor even intended for, quantitative affinity ranking. Additionally, as stated earlier, the community of researchers working specifically on predicting protein‐ligand complexes and their affinities, or the latest Boltz‐2 model purportedly capable of modeling complexes and predicting affinities, was not well represented among CASP16 predictors.

## 5 Modeling of Nucleic Acids and Their Complexes

Nucleic acid (NA) structures, particularly RNA structures, are poorly covered in the PDB, with growing experimental efforts [36, 37, 38] that would benefit enormously from better computational predictions. CASP16 featured its largest‐ever set of NA targets, including DNA and RNA monomers, NA‐NA multimers, and NA‐protein/ligand complexes [14]. This allowed for the deepest‐ever evaluation of structure prediction systems at modeling this very important class of molecules and complexes.

### 5.1 What's Currently Possible and What Isn't?

CASP16 showed solidly that NA structure prediction accuracy lags significantly behind that for proteins. No predictions of previously unseen natural RNA structures achieved a TM‐score above 0.8, a threshold considered to point at well‐defined structures [14]. This disparity highlights that strategies successful for proteins have not yet translated effectively to NAs, which still seem to be stuck at a “pre‐AlphaFold era.” Like for proteins before AF2 came out, accuracy in NA modeling is highly dependent on the availability of closely related 3D structural templates in the PDB, and there is little to no hope for accurate modeling of targets lacking templates [14].

Unfortunately, then, compared to previous CASP editions [39] or RNA‐Puzzles [18, 40] challenges, CASP16 did not show a notable increase in overall NA modeling accuracy. The good point is that the large number of targets available, covering various types of complexes, allowed for a clear investigation of what works and what doesn't, as detailed in the assessors' report [14] and also by the structure providers themselves who tested how well the best models could have (not) replaced the experimental structures [41].

Detailed features on NA pseudoknots, singlet Watson‐Crick base pairs, non‐canonical pairs, and specific tertiary motifs such as A‐minor interactions (a type of tertiary interaction where an adenine residue interacts with the minor groove of a nearby RNA helix) are all very hard to model, which is a pity because often they are at the center of special functions, as the structure providers indicated [41]. And given that coarser details about the 3D topology are so poorly predicted, it is at the moment somewhat pointless to consider these subtle structural features.

Predicting stoichiometry for RNA multimers (in Phase 0, as commented earlier in the section of protein‐containing multimers) was very poor. Even with known stoichiometry, symmetry prediction was challenging. RNA–RNA interface prediction was generally largely inaccurate, except when very close templates were available. Likewise, modeling of NA‐protein complexes also remains difficult, as discussed in Section 3.3. Good predictions rely largely on templates for both the protein and NA components, and for their interface. Finally, a few CASP16 targets entailed complexes between NAs and small molecule ligands. For ZTP‐riboswitches where good RNA and ligand pose templates existed, some groups made quite accurate predictions, but they were human participants. Meanwhile, for novel NA‐ligand targets, poor NA structure prediction precluded accurate ligand pocket modeling.

On a positive note, the secondary structures (base‐pairing patterns) predicted by the 3D modeling groups in CASP16 were often quite accurate, even when the whole 3D topology was rather wrong, in many cases outperforming dedicated secondary structure prediction algorithms that do not build 3D models. This is potentially useful for those in need of RNA secondary structure assignments only.

In summary, *de novo* prediction of NA structures as reliable as for proteins is currently not feasible, especially for targets lacking templates and with little or no hope for complex topologies as well as for non‐canonical details. The best chance for a useful model comes from homology modeling if a suitable 3D template exists, possibly with Yang‐Server or AF3 but carefully checking against possible templates. However, of potential interest for certain applications such as predicting RNA secondary structure only, 3D prediction methods can provide valuable insights, even if the 3D model itself is not very good.

### 5.2 Recommended Servers and Their Advantages Over AF3

Seven predictor groups performed above the baseline AF3, of which only Yang‐Server is a server, and all others are human groups. The differences compared to the baseline are, however, rather small, so the servers are probably just the way to go, provided good templates are available for the NA parts of the modeling. Note that a major limitation of AF3 (at least its initial version/server) is that it does not perform template searches for nucleic acids, hindering its performance on template‐amenable targets; the assessors suggested that human predictors, and even the automated Yang‐Server, may be better able to use template information [14].

### 5.3 Tips and Tricks From Top‐Performing Groups

The best chance for a useful model of an assembly made of or containing NAs comes from homology modeling if a suitable 3D template exists, possibly with Yang‐Server or AF3 but carefully checking against possible templates. As mentioned for AF3, it does not search for NA templates; however, knowing that a given PDB structure of a NA was seen by it during training means that it will inherently “use it” upon prediction.

Confidence metrics from NA or protein‐NA complex modeling should then be complemented by metrics that quantify the similarity between the target/s and any potential templates found in the PDB.

## 6 Key Practical Takeaways: Biomolecular Structure Prediction Applying CASP16's Insights

CASP16 has once again provided an invaluable snapshot of the capabilities and limitations of current biomolecular structure prediction. The profound impact of AlphaFold and related AI methodologies continues to unfold, democratizing access to state‐of‐the‐art modeling tools. We have focused here on servers and software used as provided; however, results from human groups show that expert intervention can often redound in better models—a power that, of course, isn't captured by any software and can only be harnessed via collaboration with these human experts. For more details about lab‐specific expertise, as well as for details on automated predictions, the reader is referred to the original CASP16 assessment papers [14, 15, 16, 17, 18].

### 6.1 The Rise of AlphaFold3

For many users, especially those seeking a balance between user‐friendliness and predictive power, AF3 emerges from CASP16 as a highly recommended option because it provides easy access to modeling capabilities that are at or very close to the state of the art. In particular, through its server version, which allows even users with limited computational skills to generate reasonably good models. Other AI models that can handle multiple molecule types, such as RoseTTAFold‐AllAtoms [10], Chai‐1 [13], Boltz‐2 [12] or the upcoming RosettaFold‐3 [42] (usable online at tailored websites or through services like Tamarind Bio's) might possibly be at or close to AF3, but they have not been thoroughly tested in CASP. CASP's own runs of regular AF2 through ColabFold and AF3 server as baselines for CASP16 show that naïve usage of AF3 provides close to state‐of‐the‐art (be it good or bad) predictions along all tracks, as detailed throughout this review and also (with some more caveats analyzed) by Elofsson [43].

Before delving into details, it is important to summarize why AF3 can't still provide a comprehensively reliable solution to all molecular structure prediction tasks. First, domain folding is nearly solved but certain sequences can still remain difficult, particularly those with very shallow MSAs such as viral proteins or when they make part of very intricate complexes. Next, for protein complexes the problem of structure prediction is still clearly not solved, with templates, MSAs, and stoichiometry information all being very important to improve the accuracy of models. For nucleic acids, the field is still “stuck in a pre‐AF era,” where templates are absolutely needed for even moderately good modeling. Finally, for ligands, a similar situation holds, because despite AF3 doing a reasonable job in CASP16 even for targets that lacked good templates or similar small molecules in the PDB, the benchmarking was very limited and follow‐up works showed that the model appears to rely too much on memorization, as discussed.

### 6.2 AlphaFold3 Pros and Cons

AF3's main strength lies in its ability to model a wide range of biomolecules, including proteins, nucleic acids, and ligands. The AF3 server stands out with its simple interface, fast run times, and detailed outputs, including comprehensive confidence metrics, as we present in Figure 1 (website screenshot) and in Figure S1 (custom, more complete analysis of the same run). Moreover, in CASP16, AF3 demonstrated a small but noticeable advantage over AF2, particularly in its confidence estimation and model selection capabilities.

AF3's main disadvantage is its restrictive license, which does not allow for commercial use, but this wouldn't be, in principle, a major problem for fundamental research. More importantly for academic groups, the server version of AF3 is capped in the number of daily submissions, doesn't allow the use of custom templates, and offers a tiny set of ligands. The downloadable version, however, breaks free from these limitations, offering—at the expense of ease of use—greater flexibility in terms of inputs, execution control, the possibility to fit larger molecular systems if hardware allows, and, of course, a number of runs only limited by the user's own resources. With such flexibility, one can optimize MSAs (though assessors noted this was perhaps slightly less critical than in CASP15), provide custom templates, carry out large numbers of runs with different seeds to enhance conformational sampling, and include absolutely any kind of ligand.

Recapping on AF3's limitations, the prediction of complex macromolecular assemblies, while advancing, still presents significant hurdles, particularly for highly intricate systems, in the absence of good templates for modeling and for systems involving nucleic acids. The critical role of experimental information, such as stoichiometry for complexes or 3D templates for nucleic acids, was starkly highlighted. Specialized areas like antibody–antigen docking (where some traditional docking methods still hold an edge) and protein‐ligand pose prediction (where AF3 shows immense promise as a co‐folding method) are evolving rapidly, but there is space for further improvement. All these limitations are, however, largely shared with all alternative systems. Additionally, the possibility to force post‐translational modifications in AF3 is enticing, but hasn't been thoroughly tested and seems limited according to certain reports [44]. Second, AF3 does not perform template searches for NAs, hindering its performance on template‐amenable targets—where other tools and even homology modeling could perform better, especially if the template wasn't in the PDB by the time AF3 was trained. Regarding ligand binding, as mentioned in that section, two independent broader studies showed that AF3 seems to rely largely on memorization, without much capability to understand the actual protein‐ligand interactions or perform good predictions for systems that are too far from those used in training.

### 6.3 The Relevance of Confidence Metrics, and What AlphaFold3 Provides as Such

**FIGURE 2:**
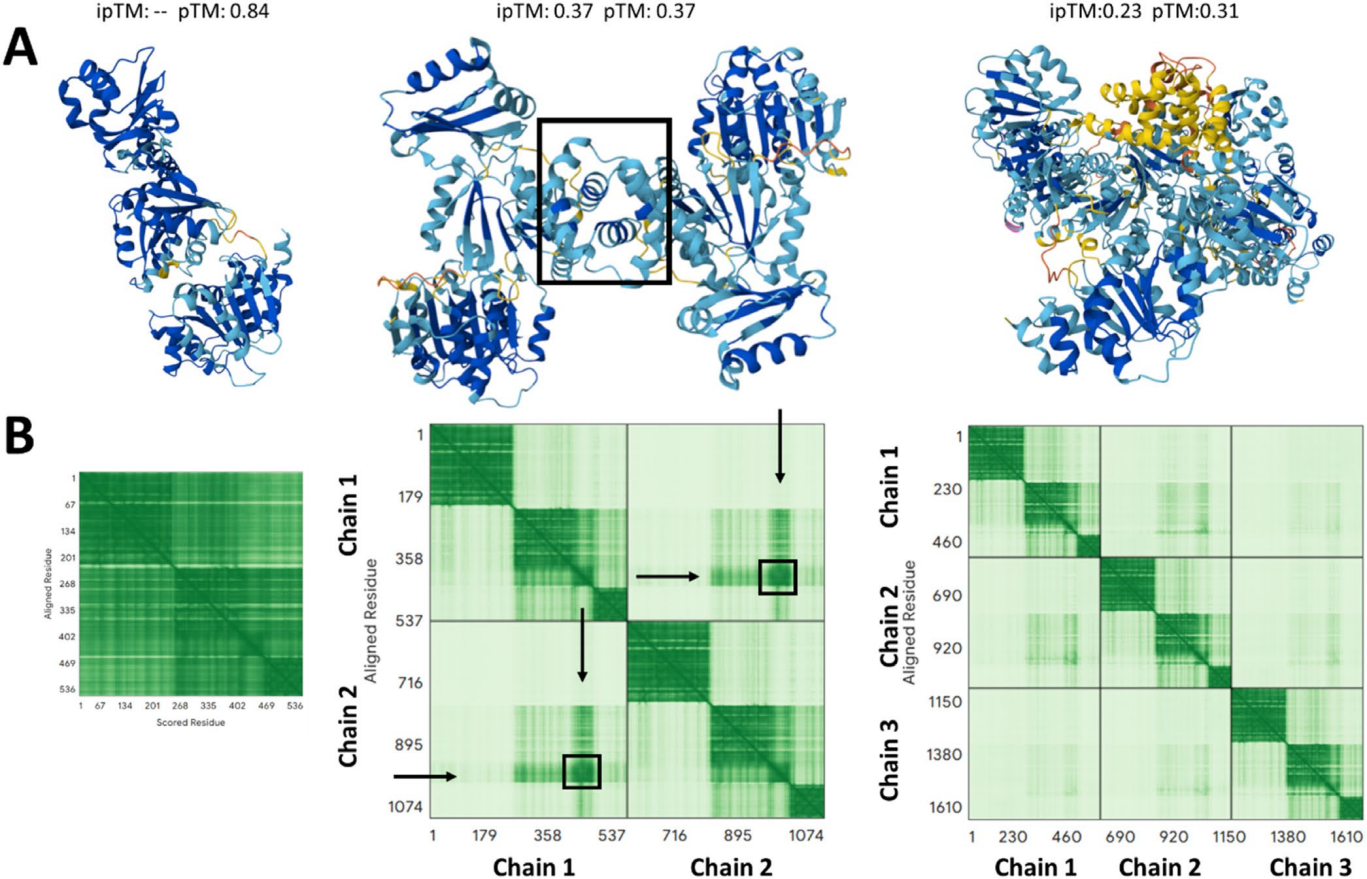
Confidence scores when modeling a protein homodimer as such, or as monomer or as trimer. Marcaida et al. [47] reported through solution experiments that the DDX21 RNA helicase is a homodimer in solution, and explored the dimerization interface via regular homology models in ways that AF2, AF3 and their metrics would have allowed much faster and reliably (an exercise actually done with AF2 for that paper upon its revision). (A) Screenshots of the models produced by AF3 server when fed 1, 2, or 3 copies of DDX21 (disordered regions left out for clarity), together with their ipTM and pTM scores. By itself, the monomer is modeled very reliably (left) but of course there's no information about possible interactions across subunits. As a complex, the global pTM and ipTM scores are on the low side but they are higher for the dimer (center); besides, with a stoichiometry of 3 one of the monomers obtains very poor pLDDT (right, subunit largely in yellow), all consistent with the protein being likely a dimer if oligomerizing, and providing potential contacts to be studied by, for example, mutagenesis. (B) The pAE matrices for the dimeric and trimeric models show a much stronger, clearer signature of interaction across subunits for the dimer than for the trimer; this corresponds to the box in panel A center (dimeric model) and is exactly where Marcaida et al. report mutants that disrupt oligomerization as probed by solution state experiments. Note that although the ipTM scores are rather poor, the pAE matrix amplified exactly where the highly confident dimerization contacts are expected.

AF3 provides global pTM scores, per‐residue and per‐atom confidence scores in the form of residue‐ or atom‐level pLDDT, residue‐residue confidence metrics (pAE), and confidence metrics specific for interfaces (ipTM). Briefly, pTM attempts to summarize the overall quality of a model in a single number that ranges from 0 to 1 (higher is better, with under 0.4 being bad and above 0.8 being good, in rough terms). Similarly, ipTM attempts to quantify in a single number the quality of all interfaces. The pLDDT score allows users to quickly assess the reliability of different regions in the model, for example of a loop relative to the core fold or of atoms in a sidechain relative to the backbone, thus highlighting potentially problematic and/or flexible loops and disordered regions. pLDDT also ranges from 0 to 1; AF3 gives a residue‐level pLDDT, mainly addressing secondary and tertiary conformation, and atom‐level pLDDT, meant to assess the quality of sidechains and ligands. Last, pAE takes the form of a matrix running through all residues and ligands modeled, attempting to measure the reliability of contacts, distances, and orientations between pairs of residues/ligands. Contrary to all other metrics which range from 0 to 1 with higher values being better, the pAE is akin to an RMSD and as such it means that lower values are better. For more details on the various quality metrics provided by AF3, the reader is referred to Figure 1, Figure S1, and the official AF3 server FAQ page. Figure 2 in turn, covers the joint use of confidence metrics with testing different stoichiometries upon complex modeling.

Running the prediction protocols with multiple seeds to generate structural variability to be considered downstream in the framework of what is already known, expected, or more sensible for the system is always a good trick to improve the modeling, as found in CASP16's track involving MassiveFold [29]. However, this study also finds that confidence metrics are not good enough by themselves to always identify the best model; therefore, critical inspection is always recommended. Practically, for end users, this is not possible on any automated server but only by using a local AF3 installation.

It is important to keep in mind that since model ranking remains a general weakness, confidence metrics can still help to easily flag obviously wrong regions. This is useful when considering multiple models, especially when extensive sampling is used, to narrow down the number of models that will be subject to deep expert inspection. Throughout this paper, we have discussed when each confidence metric should be considered; we provide here a brief comprehensive recap. The pAE matrix is especially useful for estimating the quality of domain‐domain arrangements in proteins as well as protein–protein, protein–NA, and protein‐small molecule interfaces. Atom‐wise pLDDT can help to specifically tell if sidechains and small molecules are likely correctly modeled or rather not. pTM serves as a single‐number, global estimator of model quality, while ipTM serves to estimate in a single number the accuracy of the interfaces in a complex—to be dissected individually with pAE plots as well as with ipTMs provided for all possible pairs of molecules (exemplified in Figure 2 together with the impact of providing known stoichiometries). We note at this point that ipTM has been found to have some problems, that a dedicated study proposed how to correct [22]. Finally, we stress once more that ultimately, users should always critically analyze results in light of the confidence scores, exploit biological sense to guide modeling and filter models, and leverage any available experimental data or information when possible—a front with interesting examples and pipelines already reported [45, 46].

## 7 Concluding Remarks

The prediction of complex macromolecular assemblies, while advancing, still presents significant hurdles, particularly for highly intricate systems, in the absence of good templates for modeling and for systems involving nucleic acids. The critical role of experimental information, such as stoichiometry for complexes or 3D templates for nucleic acids, was starkly highlighted. Specialized areas like antibody–antigen docking (where some traditional docking methods still hold an edge) and protein‐ligand pose prediction (where AF3 shows immense promise as a co‐folding method) are evolving rapidly, but still need major improvements. CASP16 showed that while AI tools and web‐based implementations have democratized access to state‐of‐the‐art, easy‐to‐use modeling tools, it is still important to count on expert interpretation, understanding of method‐specific limitations, and critical evaluation of confidence metrics.

Finally, for the broader community to benefit from advances demonstrated in CASP, it is crucial that participants share their code and workflows.

## Author Contributions

**Luciano A. Abriata:** conceptualization, visualization, writing – original draft, writing – review and editing. **Matteo Dal Peraro:** conceptualization, writing – original draft, writing – review and editing, validation.

## Supporting information


**Figure S1:** Dive into the example of assembly modeling with AlphaFold 3 and of its confidence metrics, from Figure 1. (A) Results of modeling the complex shown in Figure 1 of the main text, where the components are colored by molecule type. The assembly includes a peripheral membrane protein (Golph3, blue cartoons) with a palmitoylated cysteine (blue spheres), a short integral membrane helical protein followed by an unstructured region (LCS, orange) that interacts with the peripheral membrane protein, and 50 lipid molecules included for context (gray), that the program spontaneously assembled into a bilayer‐like structure that reflect the true nature of this complex. (B) Atom‐wise pLDDT traces by chain (higher is better). For proteins and nucleic acids, pLDDT is most often averaged per residue and color‐mapped onto a cartoon representation of the 3D model as shown in the inset (red is low pLDDT, blue is high pLDDT; the palmitoylated cysteine and the lipids are colored by pLDDT mapped at atomic level as clearly seen in the zoom). (C) PAE plot quantifying how reliably each residue was modeled relative to all others in the model (lower is better). Molecular graphics in this figure were rendered with PyMOL 0.99 and the plots were generated from the raw AF3 server outputs with a custom tool available at https://go.epfl.ch/af3scores.

## Data Availability

Data sharing not applicable to this article as no datasets were generated or analyzed during the current study.
