# Supplementary material for: Practical Outcomes From CASP16 for Users in Need of Biomolecular Structure Prediction
Source: Proteins. 2025 Oct 15;94(1):435–46. doi: 10.1002/prot.70078 (PMC12750028; doi:10.1002/prot.70078)
Supplement: Supplementary file 1 — Figure S1: Dive into the example of assembly modeling with AlphaFold 3 and of its confidence metrics, from Figure 1. (A) Results of modeling the complex shown in Figure 1 of the main text, where the components are colored by molecule type. The assembly includes a peripheral membrane protein (Golph3, blue cartoons) with a palmitoylated cysteine (blue spheres), a short integral membrane helical protein followed by an unstructured region (LCS, orange) that interacts with the peripheral membrane protein, and 50 lipid molecules included for context (gray), that the program spontaneously assembled into a bilayer‐like structure that reflect the true nature of this complex. (B) Atom‐wise pLDDT traces by chain (higher is better). For proteins and nucleic acids, pLDDT is most often averaged per residue and color‐mapped onto a cartoon representation of the 3D model as shown in the inset (red is low pLDDT, blue is high pLDDT; the palmitoylated cysteine and the lipids are colored by pLDDT mapped at atomic level as clearly seen in the zoom). (C) PAE plot quantifying how reliably each residue was modeled relative to all others in the model (lower is better). Molecular graphics in this figure were rendered with PyMOL 0.99 and the plots were generated from the raw AF3 server outputs with a custom tool available at https://go.epfl.ch/af3scores. [file PROT-94-435-s001.docx]

Supplementary information for

**Practical outcomes from CASP16 for users in need of biomolecular structure prediction**

***Running title: Practical outcomes from CASP16***

*Luciano A. Abriata* and Matteo Dal Peraro*

Laboratory for Biomolecular Modeling and Protein Structure Core Facility, School of Life Sciences, École Polytechnique Fédérale de Lausanne (EPFL) and Swiss Institute of Bioinformatics, CH-1015 Lausanne, Switzerland

* [luciano.abriata@epfl.ch](mailto:luciano.abriata@epfl.ch)


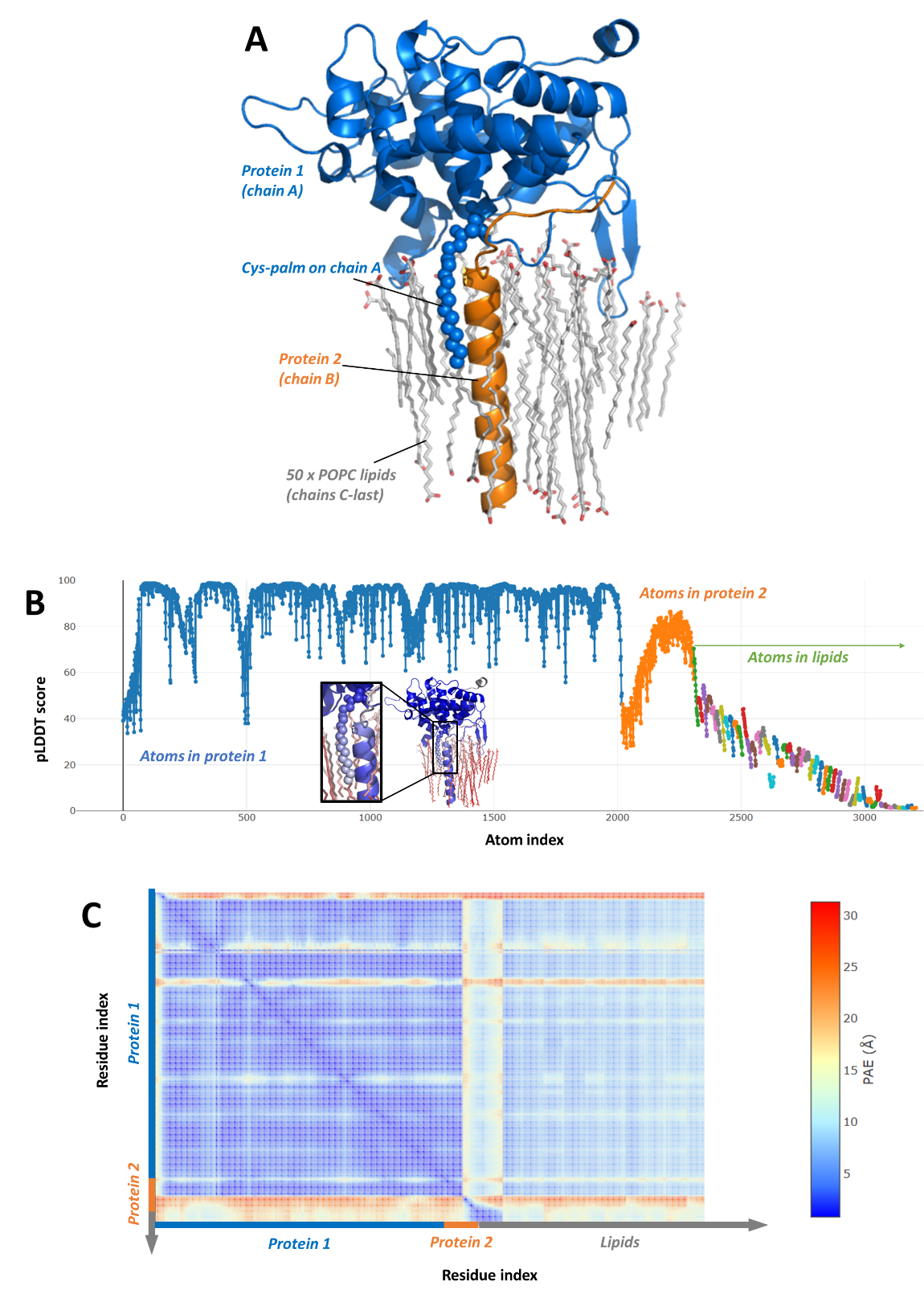


**Figure S1. Dive into the example of assembly modeling with AlphaFold 3 and of its confidence metrics, from Figure 1.** (A) Results of modeling the complex shown in Figure 1 of the main text, where the components are colored by molecule type. The assembly includes a peripheral membrane protein (Golph3, blue cartoons) with a palmitoylated cysteine (blue spheres), a short integral membrane helical protein followed by an unstructured region (LCS, orange) that interacts with the peripheral membrane protein, and 50 lipid molecules included for context (grey), that the program spontaneously assembled into a bilayer-like structure that reflect the true nature of this complex. (B) Atom-wise pLDDT traces by chain (higher is better). For proteins and nucleic acids, pLDDT is most often averaged per residue and color-mapped onto a cartoon representation of the 3D model as shown in the inset (red is low pLDDT, blue is high pLDDT; the palmitoylated cysteine and the lipids are colored by pLDDT mapped at atomic level as clearly seen in the zoom). (C) PAE plot quantifying how reliably each residue was modeled relative to all others in the model (lower is better). Molecular graphics in this figure were rendered with PyMOL 0.99 and the plots were generated from the raw AF3 server outputs with a custom tool available at <https://go.epfl.ch/af3scores>.
